# First Lines of Physiology; Designed for the Use of Students of Medicine

**Published:** 1842-01

**Authors:** 


Art. XII.—*First Lines of Physiology; designed for the Use of Students of Medicine.* By Daniel Oliver, m.d., Professor of Materia Medica in the Medical College of Ohio.—*Philadelphia,* 1840. 8vo, pp. 514.

We noticed with commendation (Br. and For. Med. Rev., vol. VI. 195,) this work on its first appearance. We have only room now to state that it is much enlarged and greatly improved, and cannot fail to be highly useful to the author's countrymen, "especially to those for whose use it was originally designed, who are preparing to enrol themselves among the members of the medical profession."

